# Mnemonic of Principles for Effective Specialist–Generalist Conferences: The BRIDGE

**DOI:** 10.1002/jgf2.70113

**Published:** 2026-03-19

**Authors:** Yohei Kanzawa, Yayoi Tetsuou Tsukada, Kenta Ishibashi, Masataka Ono, Yui Matsushima, Motohiro Shingu, Hideki Saito, Koichi Tamita, Naoto Ishimaru

**Affiliations:** ^1^ Department of General Internal Medicine Kobe University Hospital Kobe Hyogo Japan; ^2^ Department of General Internal Medicine Akashi Medical Center Akashi Hyogo Japan; ^3^ Department of ER and General Medicine Nippon Medical School Musashi‐Kosugi Hospital Kawasaki Kanagawa Japan; ^4^ Department of General Medicine and Health Science Nippon Medical School Bunkyo‐ku Tokyo Japan; ^5^ Department of Cardiovascular Medicine Akashi Medical Center Akashi Hyogo Japan; ^6^ Department of Cardiology Seirei Hamamatsu General Hospital Hamamatsu Shizuoka Japan

Heart failure is a major public health issue, with an especially high burden in rapidly aging societies. As patients with heart failure increase, many present with multiple comorbidities, frailty, and complex social contexts, complicating their management. Comprehensive and context‐oriented care provided by generalists is thus important in coordinating multidisciplinary management while addressing patients' overall health status and quality of life [1]. Close collaboration between generalists and specialists, who contribute disease‐specific expertise, is important in providing high‐quality care for patients with multimorbidity. Conferences for information sharing and joint decision‐making are important for effective teamwork in places where this collaboration is required. For example, we hold weekly heart failure conferences at our hospital. They include discussion with cardiovascular specialists about all patients admitted to the Department of General Internal Medicine with heart failure, and it is thought to contribute to improved quality of care.

Here, we propose “BRIDGE” (Figure 1), a practical mnemonic framework for successful generalist–specialist conferences based on our experience and discussions held during a recent symposium.

**FIGURE 1:**
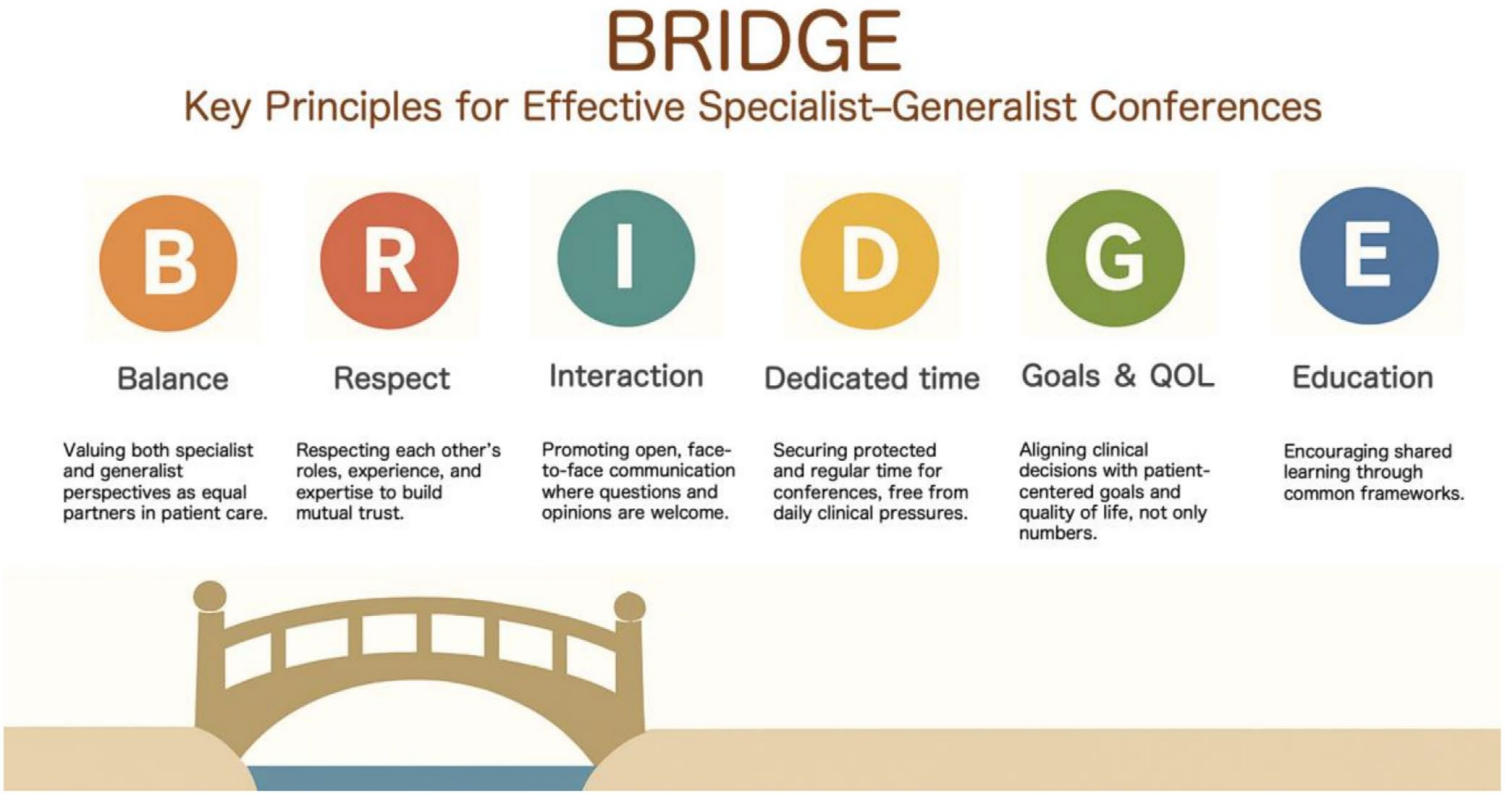
The BRIDGE framework for effective conferences between specialists and generalists. Together, these elements function as a “bridge” that supports collaborative, patient‐centered discussions in multidisciplinary clinical conferences. BRIDGE: A mnemonic for effective conferences between specialists and generalists.

“*B*alance” emphasizes valuing both generalist and specialist perspectives equally, recognizing that neither discipline should dominate, and that patient care is best supported by working cohesively. Collaboration across professional boundaries reportedly improves clinical outcomes [2]. “*R*espect” highlights the importance of mutual respect for differing roles, expertise, and responsibilities, which enables patient‐centered discussions about the most appropriate attending physicians or department. Higher levels of mutual respect within teams have been associated with significantly lower rates of medical errors [3]. “*I*nteraction” underscores the value of face‐to‐face communication and an atmosphere that encourages questions and opinions, because regular in‐person discussions improve the quality of information sharing and reduce communication failures [4]. “*D*edicated time” refers to securing protected and scheduled time for conferences despite clinical busyness. Our conferences are held every Tuesday at 13:00, and evidence from structured interdisciplinary rounds suggests that fixed, recurring meeting times contribute to continuity, trust‐building, and improved clinical outcomes such as reduced length of stay in ICU [5]. “*G*oals and quality of life” emphasize that treatment objectives should extend beyond disease‐oriented metrics and guidelines to incorporate patients' values and preferences, social contexts, and overall quality of life. Shared goal setting among generalists, specialists, and multidisciplinary teams facilitates patient‐centered decision‐making and may also reduce clinician burden [6]. Finally, “*E*ducation” highlights shared learning through the use of common templates and mutual teaching, leading to an overall improvement in team‐based care. Generalists contribute insights into patient context and complexity, while specialists share advanced medical knowledge and treatment strategies. BRIDGE may become a practical and reproducible mnemonic framework to support effective collaboration between generalists and specialists and to enhance the quality of conferences in the era of increasing multi‐morbidity. To facilitate implementation elsewhere, we have found starting with a small, regularly scheduled conference and using a simple shared template to be effective. Potential barriers include time constraints, hierarchical culture, and unclear role definitions. Future research should evaluate whether introducing the framework is associated with improvements in clinical outcomes, interprofessional collaboration scores, or clinician well‐being across different healthcare settings.

## Author Contributions

**Yohei Kanzawa:** writing – original draft, conceptualization, visualization. **Yayoi Tetsuou Tsukada:** writing – review and editing. **Kenta Ishibashi:** writing – review and editing. **Masataka Ono:** writing – review and editing. **Yui Matsushima:** writing – review and editing. **Motohiro Shingu:** writing – review and editing. **Hideki Saito:** writing – review and editing. **Koichi Tamita:** writing – review and editing. **Naoto Ishimaru:** writing – review and editing.

## Funding

The authors have nothing to report.

## Ethics Statement

The authors have nothing to report.

## Consent

The authors have nothing to report.

## Conflicts of Interest

The authors declare no conflicts of interest.

## Data Availability

The authors have nothing to report.
